# Inwardly Rectifying Potassium (Kir) Channels Represent a Critical Ion Conductance Pathway in the Nervous Systems of Insects

**DOI:** 10.1038/s41598-018-20005-z

**Published:** 2018-01-25

**Authors:** Rui Chen, Daniel R. Swale

**Affiliations:** Louisiana State University AgCenter, Department of Entomology, Baton Rouge, LA 70803 USA

## Abstract

A complete understanding of the physiological pathways critical for proper function of the insect nervous system is still lacking. The recent development of potent and selective small-molecule modulators of insect inward rectifier potassium (Kir) channels has enabled the interrogation of the physiological role and toxicological potential of Kir channels within various insect tissue systems. Therefore, we aimed to highlight the physiological and functional role of neural Kir channels the central nervous system, muscular system, and neuromuscular system through pharmacological and genetic manipulations. Our data provide significant evidence that *Drosophila* neural systems rely on the inward conductance of K^+^ ions for proper function since pharmacological inhibition and genetic ablation of neural Kir channels yielded dramatic alterations of the CNS spike discharge frequency and broadening and reduced amplitude of the evoked EPSP at the neuromuscular junction. Based on these data, we conclude that neural Kir channels in insects (1) are critical for proper function of the insect nervous system, (2) represents an unexplored physiological pathway that is likely to shape the understanding of neuronal signaling, maintenance of membrane potentials, and maintenance of the ionic balance of insects, and (3) are capable of inducing acute toxicity to insects through neurological poisoning.

## Introduction

The establishment of insecticide resistance within multiple arthropod vectors of human pathogens has been, at least in part, the driving force behind the prolific advancement of the fields of insecticide science and insect molecular physiology. The goal of mitigating the various resistance mechanisms has been a multidisciplinary and transdisciplinary approach that has resulted in a detailed understanding of molecular genetics, transcriptomics, biochemistry, cellular physiology, and neuroendocrinology of non-model insects, such as mosquitoes. In addition to these fields, the reduced efficacy of currently approved classes of insecticides has dramatically increased interest of identifying novel molecular targets for insecticide design^1–5^ and/or development of novel chemical scaffolds targeting previously exploited proteins^6–9^. A variety of new target sites and chemical scaffolds have been identified and characterized in the past decade that include transient receptor proteins^5^, G-protein coupled receptors^10^, dopaminergic pathways^4^, and K^+^ ion channels^1–3,11^.

Inward rectifier potassium (Kir) channels belong to a large ‘superfamily’ of K^+^ ion channels that includes the voltage-gated, two-pore, calcium-gated, and cyclic nucleotide-gated channels^12,13^. Kir channels function as biological diodes due to their unique ability to mediate the inward flow of K^+^ ions at hyperpolarizing membrane voltages more readily than the outward flow of K^+^ at depolarizing voltages. On a molecular level, Kir channel are structurally simple ion channels that consists of 4 subunits assembled around a central, water-filled pore, through which K^+^ ions move down their electrochemical gradient to traverse the plasma membrane. Each subunit consists of a central transmembrane domain, a re-entrant pore-forming loop, and a cytoplasmic domain comprised of amino and carboxyl termini^14^.

Recent genetic and pharmacological evidence suggests that Kir channels could represent viable targets for new insecticides. In *Drosophila melanogaster*, embryonic depletion of Kir1, Kir2, or Kir3 mRNA leads to death or defects in wing development^15^. Reduction of Kir1 and Kir2 mRNA expression in the Malpighian (renal) tubules of *Drosophila* or inhibition of Kir channels in isolated mosquito Malpighian tubules with barium chloride (BaCl_2_) dramatically reduces the transepithelial secretion of fluid and K^+^^16,17^, indicating Kir channels expressed in the Malpighian tubules may be an exploitable insecticide target site. Considering this, high-throughput screens (HTS) of chemical libraries were performed to identify small-molecule modulators of mosquito Kir1 channels, which is the principal conductance pathway in mosquito Malpighian tubules^17^. Structurally distinct small molecules were identified (i.e. VU573, VU590, or VU625) and pharmacological inhibition of *Aedes aegypti* Kir1 was shown to disrupt the secretion of fluid and K^+^ in isolated Malpighian tubules, urine production, and K^+^ homeostasis in intact females^1,18,19^. Similarly, a Kir1 inhibitor, termed VU041, was identified in a subsequent HTS campaign and was shown to (1) be highly potent against the *Anopheles gambiae* Kir1 (ca. 500 nanomolar), (2) exhibit topical toxicity (ca. 1 μg/mosquito) to insecticide-susceptible and carbamate/pyrethroid-resistant strains of mosquitoes, (3) and display high selectivity for mosquito Kir channels over mammalian Kir channel orthologs^3^.

Previous work indicates that VU041-mediated toxicity stems from inhibition of the Kir1 channel within the Malpighian tubules to induce tubule failure and an inability to maintain K^+^ homeostatsis after blood feeding^3^. However, after exposure to lethal doses of VU041, *An. gambiae* and *A. aegypti* were found to display both hyperexcitatory and lethargic tendencies that were complexed with uncoordinated movements^3^, which is reminiscent of neurological poisoning. Furthermore, acute toxicity (ca. 1–3 hours) was observed after exposure to VU041, similar to other insecticides that poison the nervous system. Lastly, previous studies have shown that select Kir channel inhibitors were capable of inducing a flightless behavior where mosquitoes were ambulatory, yet were not able to fly, presumably due to failure of the nervous or muscular systems^2^. Although it is possible that the mortality is due to complete systems failure stemming from ubiquitous expression of Kir channels or due to accumulated waste that remains due to impaired Malpighian tubule function^3^, it is also reasonable to predict that VU041 is directly altering the functional capacity of Kir channels expressed in the nervous system to yield toxicity. Unfortunately, there have been no studies to characterize the physiological role of Kir channels in the insect nervous systems, which limits the ability to infer the toxicological potential of these neural proteins. Studies using RT-PCR have shown that the head of *A. aegypti* is enriched with Kir2B’ (vector base accession number: AEL013373) mRNA (personal communication, Dr. Peter Piermarini, The Ohio State University), suggesting that poisoning of the mosquito central nervous system (CNS) through Kir inhibition is indeed possible. Unfortunately, electrophysiological recordings of mosquito CNS activity have yet to be achieved, which limits the ability to infer the physiological role or toxicological potential of neural Kir channels of mosquitoes. However, electrophysiological recordings from an excised CNS of *D. melanogaster* is possible^20^ and further, the gene encoding Kir2, termed *irk*2, is highly concentrated in the adult head, CNS, and the thoracic-abdominal ganglia^21^. This suggests that *D. melanogaster* may represent a suitable substitute for mosquitoes and will enable the characterization of the physiological role Kir channels have in the insect nervous system.

Considering (1) the foundational role of Kir channels in mammalian and insect cellular physiology, (2) deletion of *irk*2 gene in *Drosophila* is homozygous lethal^22^, (3) the signs of intoxication after exposure to Kir channel modulators being reminiscent of neurological poisoning, and (4) the overexpression of Kir mRNA in mosquito and *Drosophila* neural tissues, we hypothesized that Kir channels regulate neuronal signaling and excitability of insect nervous systems and are a critical conductance pathway for proper functioning of the insect nervous system. Therefore, the goals of the present study were to employ electrophysiological methods combined with genetic and pharmacological techniques to determine the physiological importance of Kir channels in insect CNS, neuromuscular junction, and muscular systems that will provide insight into targeting neural Kir channels as a novel insecticide target site. Additionally, data collected in this study begin to bridge the fundamental knowledge gap regarding unexplored physiological pathways in the insect nervous system that will provide a more holistic understanding to neuronal excitability and neurotransmission of insects.

## Methods

### Insect Stocks and Rearing Conditions

Four strains of *D. melanogaster* were used in this study. The wildtype Oregon-R (OR) strain was provided by Dr. Jeffrey Bloomquist at the University of Florida and was originally donated by Doug Knipple, Cornell University, Ithaca NY, USA. All GAL4-UAS fly strains were purchased from Bloomington Drosophila Stock Center (Bloomington, IN, USA). The GAL4-UAS strain 3739 expresses the Gal-4 pattern in the brain of 3^rd^-instars with strong expression throughout the CNS, but in the disks. The strain 41981 expresses dsRNA for RNAi of Kir2 (i*rk2*) under UAS control. The strain 41554 expresses hairpin RNA (hpRNA) under the control of UAS for RNAi of GFP and was used as a negative knockdown control. The genotypes of each strain are as follows: 3739, P(w[+mW.hs] = GawB)c698a, w[1118]; 41981, y[1] sc[*] v[1]; P(y[+t7.7] v[+t1.8] = TRiP.HMS02379)attP2; 41554, y[1] sc[*] v[1]; P(y[+t7.7] v[+t1.8] = VALIUM20-EGFP.shRNA.2)attP2.

All fly strains have been maintained in culture at the Louisiana State University since April 2015 and were reared on standard medium in *Drosophila* tubes at 25 °C, 12·hour-12·hour photoperiod and 55% relative humidity. For dissection, flies were anaesthetized by chilling on ice and decapitated before dissecting out CNS in Schneider’s medium (Invitrogen, Paisley, Scotland, UK).

### Chemicals

**Figure 1:**
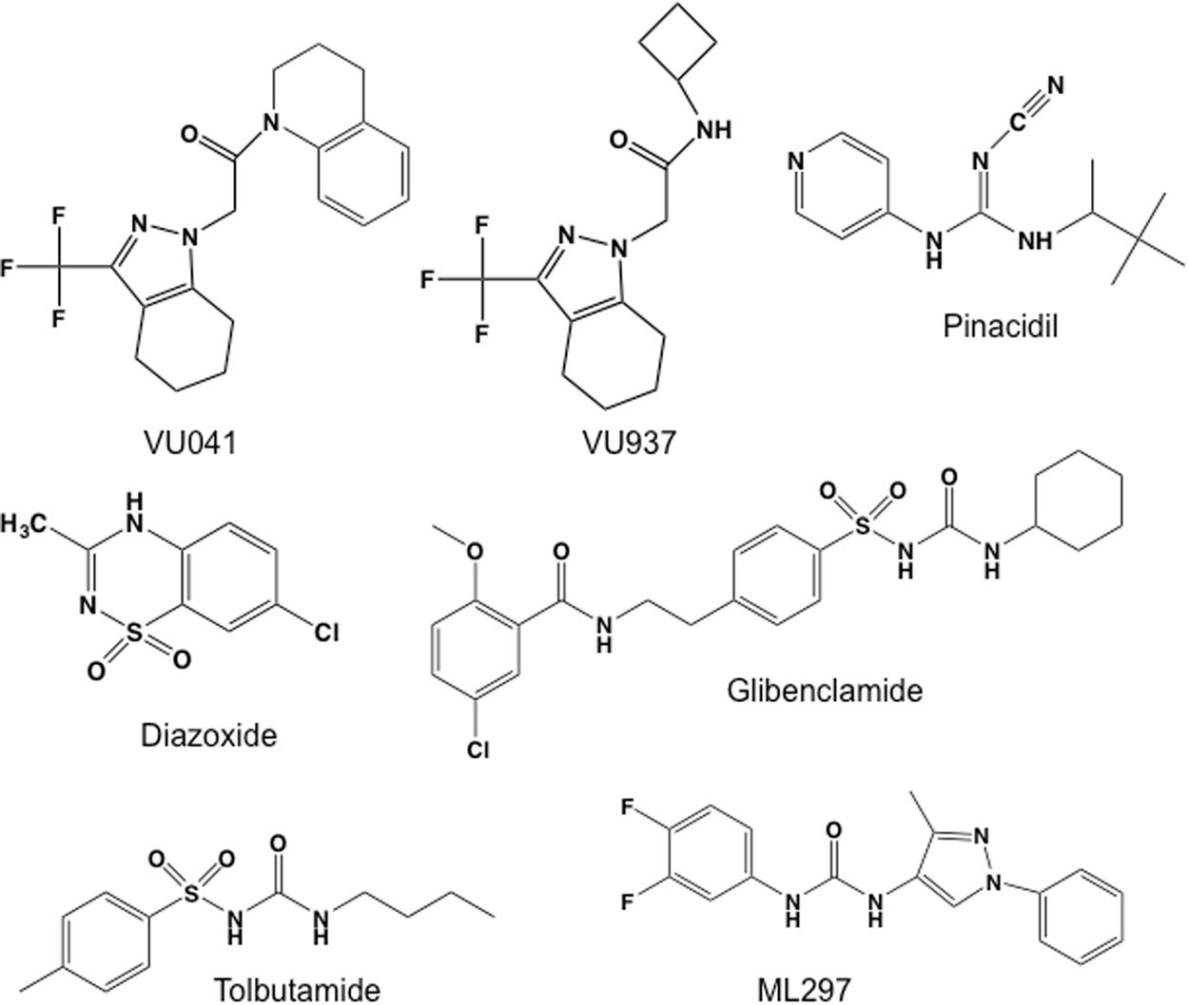
Chemical structures of Kir channel inhibitors used in this study.

The Kir channel inhibitor VU041 and the inactive analog VU937 were originally discovered in HTS against the *Anopheles gambiae* Kir1 channel^3^. Both compounds were synthesized by Dr. Corey Hopkins at the Vanderbilt Center for Neuroscience Drug Discovery using methods described in Swale *et al*.^3^. ML297, pinacidil, glybenclamide, tolbutamide, and diazoxide were purchased from Sigma-Aldrich (St. Louis, MO, USA). Chemical structures of the modulators used in this study are shown in Fig. 1.

### Video-tracking software and recordings

EthoVision® XT video recording software was used for recording the movements of adult *Drosophila* flies exposed to VU041 (Noldus, Leesburg, VA). The wings of each fly were removed with scissors immediately above the wing joint to prevent movement in the z-axis, which would skew recording measurements. We did not observe any hemolymph loss or mortality after removal of the wings. Adult female flies were injected with 25 nL of VU041, VU937, or PBS (control) and were held in a chamber at 25 °C for 60 minutes. After the incubation period, individual flies were transferred to a 100 mm × 20 mm culture dish that had a white filter paper on the bottom of the dish to increase contrast. Flies were given 3 minutes to become acclimated to the dish, lights, etc. and the recording was performed for a total of 30-seconds to record total distance traveled. The average (n = 30) distance traveled per fly was calculated by the EthoVision® video-tracking software and the data were statistically analyzed using a one-way ANOVA with a multiple comparisons test to determine significantly different means in GraphPad Prism (La Jolla, CA) software.

### Electrophysiological Studies of Drosophila melanogaster neural systems

Suction electrode electrophysiological recordings were performed on the CNS of 3^rd^-instar *D. melanogaster*. Glass pipette electrodes were pulled from borosilicate glass capillaries on a P-1000 Flaming/Brown micropipette puller (Sutter Instrument, Novato CA, USA). For CNS recordings [20], the CNS and descending nerves were excised from the larvae and placed in a separate dish with physiological saline (200 µL) containing: 157 mM NaCl, 3 mM KCl, 2 mM CaCl_2_, and 4 mM HEPES, pH = 7.25. The CNS was manually transected posterior to the cerebral lobes to disrupt the blood-brain barrier and enhance chemical penetration^23,24^. Peripheral nerve trunks were drawn into a recording suction electrode and electrical activity was monitored from descending nerves originating from the CNS, with amplification by an AC/DC amplifier (Model 1700, A-M Systems, Inc., Carlsborg, WA, USA). Descending electrical activity was subjected to window amplitude discrimination and converted on-line into a rate plot, expressed in Hertz (Hz), using LabChart7 Pro (ADInstruments, Colorado Springs, CO, USA). Noise (60 Hz) was eliminated using Hum Bug (A-M Systems, Sequim, WA, USA). Activity was monitored for a five minute time period to establish a constant baseline spike discharge rate, as the spike frequency typically increased from 0 to 5 minutes before stabilization. After a baseline was established, the CNS preparation was directly exposed to test compounds by adding 200 µL of solution to the bath containing 200 uL of saline. The final concentration of solvent in the bath was 0.1% DMSO. Frequencies were measured for 3–5 min for each concentration prior to the addition of the next drug concentration. Mean spike frequencies for each concentration were used to construct concentration-response curves to determine IC_50_ values. IC_50_’s were calculated by nonlinear regression (variable slope) using a Hill equation in GraphPad Prism^TM^ (GraphPad Software, San Diego, CA, USA). Each drug concentration was replicated 3–10 times.

Muscle membrane potential and neuromuscular recordings of the evoked EPSP were performed on 3^rd^-instar *D. melanogaster*, essentially as described previously^24,25^. A maggot was immobilized with pins, and the nervous and musculature systems were exposed. The saline contained 140 mM NaCl, 0.75 mM CaCl_2_, 5 mM KCl, 4 mM MgCl_2_, 5 mM NaHCO_3_, and 5 mM HEPES (pH = 7.25). The nerves were severed from the base of the CNS, which was removed. The changes in muscle membrane potential after Kir channel modulation, a recording glass capilallry microelectrode was filled with 1 M KCl and was placed in a large fiber of ventrolateral muscle. For neuromuscular junction recordings, a lateral nerve trunk innervating the longitudinal muscles was drawn into a suction electrode filled with saline. Stimuli were applied at 1 volt and of 0.2 sec duration to elicit a contraction from the longitudinal muscles. The stimulated muscle was then impaled with a recording glass capillary microelectrode filled with 1 M KCl to record effects on the evoked EPSP and membrane potential. The signals for RMP and evoked EPSPs were amplified via an Axoclamp 900A (Molecular Devices, Sunnyvale, CA, USA), before filtering through a Hum Bug noise eliminator (A-M Systems, Sequim, WA, USA) and digitized using LabChart 7 (ADInstruments PowerLab 4/30, Colorado Springs, CO, USA), which also included a 50 Hz low pass digital filter. Chemicals were applied to the preparation directly by hand pipetting 100 µL of solution into the bath volume of 100 µL. For analysis, data points describing the evoked EPSP amplitude and width of each waveform were taken at the beginning, middle, and end each treatment period. These values were averaged and treated as one replicate. A total of 8 replicates were used for control, VU041, and VU937.

### Genetic knockdown of CNS specific irk2

Advances in *Drosophila* genetics has enabled tissue specific knockdown of specific genes through the GAL4-UAS system. This technology has been used for the previous decade and is based on the properties of the yeast transcriptional activator Gal4 that activates transcription of its target genes by binding to upstream activating sequence (UAS). The GAL4-UAS construct binds next to the gene of interest, which in this case is hairpin RNA (hpRNA) for *irk*2, to genetically enhance or decrease mRNA expression^26–28^. The two components, GAL4 and UAS are carried in separate *Drosophila* stocks that allow for hundreds of combinatorial possibilities after a simple parental cross. In this study, we utilized a strain of fly that expressed the GAL4-UAS promoter only in the CNS of 3^rd^-instars, which is the lifestage analyzed using electrophysiological methods. These methods are described in Johnston (2002)^29^ and enabled the CNS-specific knockdown of the gene encoding Kir2.

Knockdown was achieved by crossing virgin females from the respective Kir2 RNAi strain (Bloomington stock 42644) with males from the CNS expressing GAL4-UAS strain (Bloomington stock 6870). The flies were given 96 hours to mate and oviposit prior to removal from the growing medium. F_1_ offspring were allowed to emerge and adults were used in the study immediately upon emergence. The genotype expression of the *irk*2 RNAi (Bloomington stock number 41981) was on the X-chromosome and therefore, male GAL4-UAS flies (3739) were crossed with virgin females from strain 41981 or 41554.

### RNA isolation, cDNA synthesis, and Quantitative-PCR

Total RNA was isolated and extracted from 30 *Drosophila* larvae CNS, whole body, or carcass using TRIzol^®^ Reagent (Life Technologies, Carlsbad, CA) and purified using the RNeasy kit (Qiagen, Valencia, CA). First-strand cDNA was synthesized from poly(A) RNA using the SuperScript^®^ III First-Strand Synthesis System for real-time quantitative PCR (qRT-PCR) (Life Technologies) according to manufacturer instructions. qRT-PCR was then performed on an Qiagen Rotor Gene Q 2Plex Real-Time PCR System using the operating instructions. Relative quantification was carried out using the 2-^DDCT^ method^30^, and beta-actin was used as the reference gene. Appropriate controls, such as DNAse and removal of reverse transcriptase, were performed to ensure the sample was not contaminated with genomic DNA. The CNS dissection included as many descending neurons as possible and the carcass was comprised of just the body wall muscle and associated neurons. All primers used in this study were purchased from Life Technologies with primer reference numbers for the *irk*1, *irk*2*, irk*3 and actin genes being Dm02143600_s1, Dm02143725_g1, Dm01796588_g1, and Dm02361909_s1, respectively. Five biological replicates were conducted and each was analyzed in triplicate. The graphed output displays average fold-change in mRNA levels relative to the wildtype Oregon-R control CNS.

## Results

### ‘Flightless’ Phenotype After Exposure to VU041

**Figure 2:**
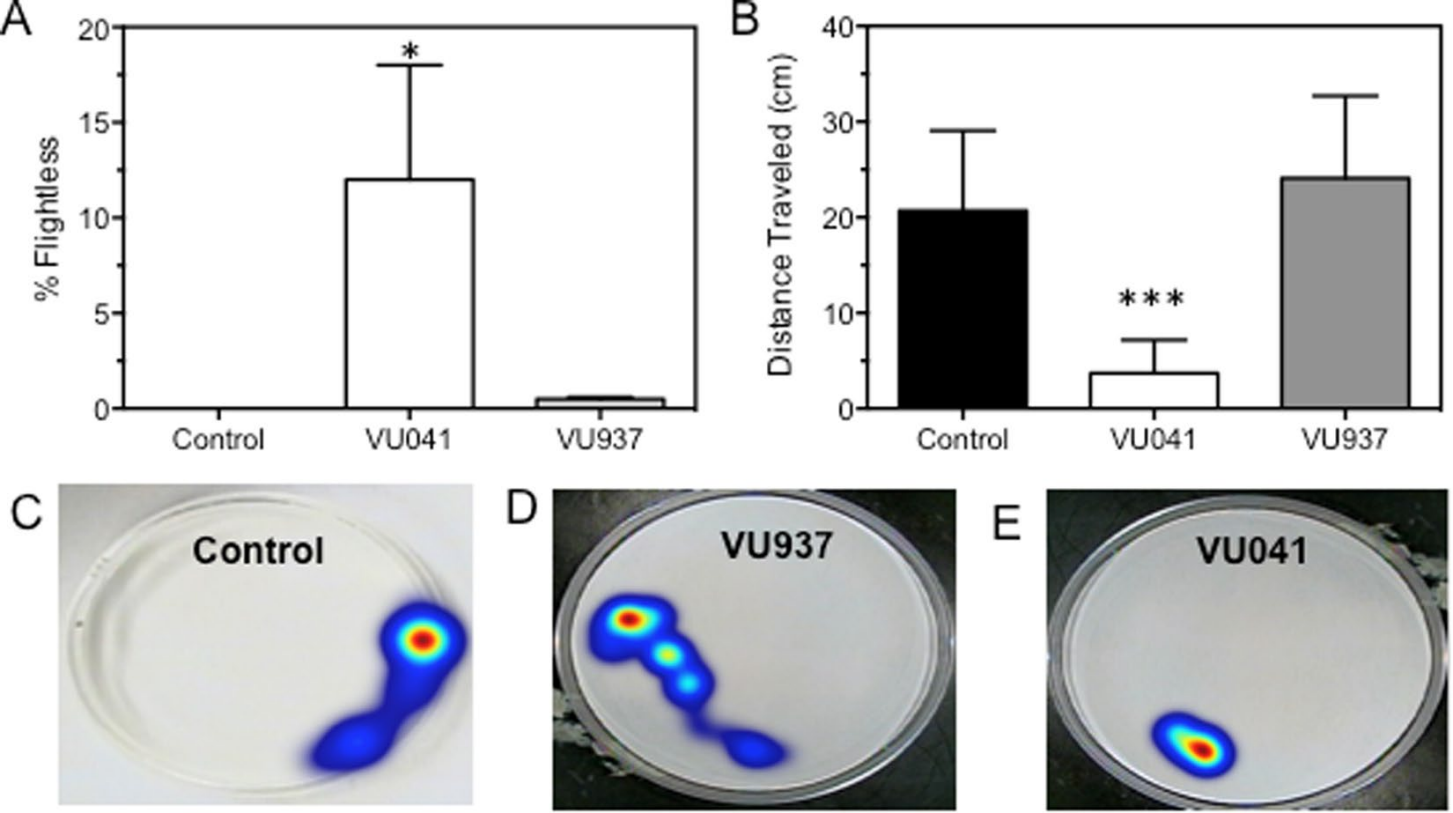
Influence of VU041 to adult *Drosophila* behavior. (**A**) Percent of injected flies that displayed the ‘flightless’ phenotype after injection with solvent control, VU041, and VU937. Bars represent mean (n = 200) and error bars represent SEM. (**B**) Total distance traveled 60-minutes after injection of solvent control, VU041, or VU937. Bars represent mean (n = 30) and error bars represent SEM. Representative heat maps of fly movements during the 30-second recording period for control and vehicle control (**C**), VU937 (**D**), and VU041 treated flies (**E**). Asterisks represent statistical significance with *representing P < 0.05 and ***representing P < 0.0001 as determined by a one-way ANOVA with multiple comparisons test.

Injection of a sub-lethal dose of VU041 (50 ng/fly) into the thorax of *D. melanogaster* where 12 ± 6% of injected flies (n = 200) were rendered flightless 3 hours post- injection, which was statistically significant (P < 0.05) from solvent control (Fig. 2A). Importantly, injection of the inactive analog, termed VU937, resulted in only 1 fly being rendered flightless out of 200 injected flies, suggesting that the flightless phenotype is indeed due to Kir channel inhibition (Fig. 2A). Interestingly, the ‘flightless’ flies were still ambulatory and would jump away from a mechanical stimulus, yet could not raise their wings to initiate flight.

### Signs of Intoxication

After treatment with lethal doses/concentrations of VU041, mosquitoes and *Drosophila melanogaster* were found to display a combination of hyperexcitation and lethargy. Approximately 20 minutes after exposure, the flies displayed hyperexcitation that was defined as twitching of legs and increased wing beat frequency. The bouts of hyperexcitation were intermixed with lethargy where the flies rested on the bottom of the holding chamber with a splayed posture and, in flies that did not display a ‘flightless’ phenotype, a slow response to mechanical stimuli. During the hyperexcitation bouts, the flies did not walk or fly around the holding chamber, which is in contrast to hyperexcitation derived from cholinergic poisoning^24^. Treated flies that did not display a ‘flightless’ phenotype responded slowly to mechanical stimuli and were still lethargic. Figure 2B summarizes the lethargic tendencies of VU041-poisoned flies. Over the 30-second recording period, control flies was found to travel 20.7 ± 8.3 cm whereas the VU041 treated flies were found to travel only 3.7 ± 3.4 cm, a statistically significant reduction (P < 0.0001). Importantly, VU937 did not influence the behavior of the flies (24.1 ± 8.6 cm) when compared to the control, suggesting that Kir inhibition results in the described signs of intoxication (Fig. 2B). Representative heat maps depicting the mobility of solvent-control, VU937, and VU041 treated flies are shown in Fig. 2C,D and E, respectively.

### Influence of pharmacological inhibition of Kir channels to CNS activity

**Figure 3:**
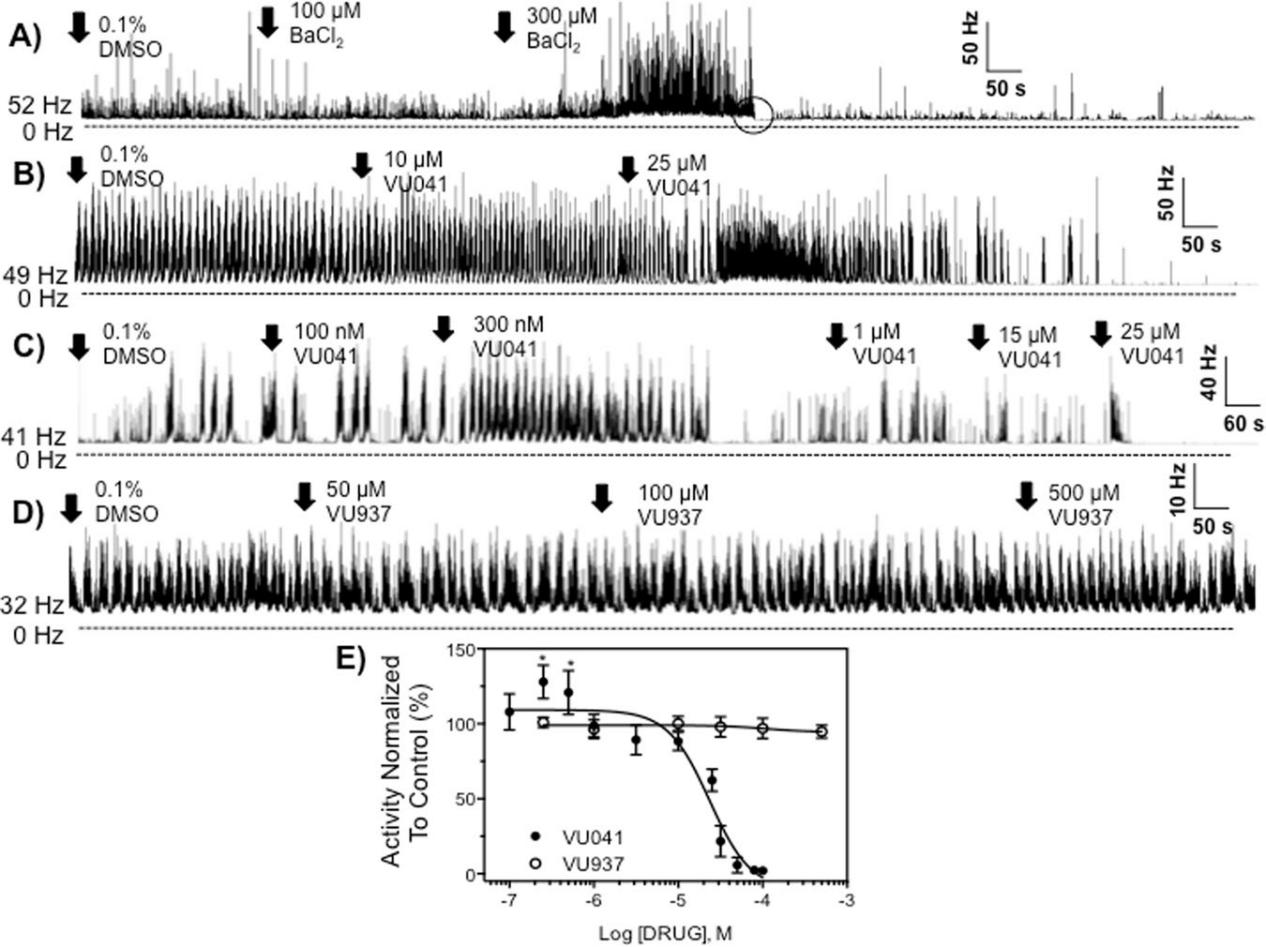
Neurophysiological recordings from the CNS of third instar larvae of *D. melanogasgter* after exposure to pharmacological modulators of Kir channels. Representative nerve discharge traces before and after exposure to (**A**) BaCl_2_, (**B**) high concentrations of VU041, (**C**) low concentrations of VU041, and (**D**) the inactive analog termed VU937. Initial spike discharge frequencies in spikes/second (Hz) for each experiment are given to the left of each trace. (**E**) Concentration-response curves for VU041 and VU937 on CNS nerve discharge of *D. melanogaster* larvae from replicated recordings (n = 3–5 concentration per curve, with each concentration replicated at least 5 times). Data points represent mean percentage increase of baseline spike discharge frequency, and error bars represent SEM of drug concentrations replicated at least 5 times. When error bars are absent, it is because they are smaller than the size of the symbol. Asterisks represent statistical significance with *representing P < 0.05 as determined by an unpaired t-test to the average baseline spike discharge frequency.

*Drosophila* larval CNS recordings were performed in an effort to test the initial hypothesis that Kir channels are an essential potassium (K^+^) ion transport pathway that mediates, at least in part, proper neurotransmission, and that VU041 is a nerve poison. To begin testing these hypotheses, the non-specific Kir channel blocker, barium chloride (BaCl_2_), was applied to the transected CNS preparation at low- to mid- micromolar concentrations. Interestingly, exposure to 100 μM BaCl_2_ yielded no alteration of the CNS spike discharge frequency whereas a 280 ± 72% increase was observed after exposure to 300 μM BaCl_2_ (Fig. 3A). After approximately 4–6 minutes of neuroexcitation, the CNS activity was spontaneously reduced (Fig. 3, trace 1, black circle) and remained in a quiescent state for the remainder of the recording. Importantly, spike discharge of the CNS was not dead since we consistently observed a firing frequency of 5–15 Hz.

To ensure the increase in CNS activity observed with BaCl_2_ was indeed due to Kir channel modulation, we explored the influence of the specific Kir channel blocker, VU041, to the *Drosophila* CNS. Exposure of the CNS to a concentration of 25 μM VU041 yielded an increase in CNS activity followed by a slow, but steady decline in spike discharge frequency, response similar to the pattern of firing observed after exposure to 300 μM BaCl_2_. Representative recordings of the spike rate in the presence of BaCl_2_ and VU041 are shown in Fig. 3A,B, where the rhythmic discharge is transformed into constant firing that subsides to near zero over the ensuing observation period. The construction of a concentration-response curve (CRC) of VU041 produced a biphasic response to the CNS activity with lower concentrations yielding an increase in CNS spike discharge frequency and higher concentrations yielding a depression of CNS activity (Fig. 3B,C). Exposure to 300 nM and 700 nM VU041 increased the spike discharge frequency by 32 ± 6% and 20 ± 8%, respectively, a statistically significant increase when compared to baseline spike discharge frequency (P < 0.05). At increasing concentrations, VU041 was found to have a depressant effect on the *Drosophila* CNS activity with a 50% inhibitory concentration (IC_50_) of 23 μM (95% CI: 17–31 μM; Hill coefficient: −1.6, R^2^: 0.93; Fig. 3E). Importantly, exposure of the CNS to the inactive analog of VU041, termed VU937, did not affect spike discharge frequency at concentrations up to 500 μM (Fig. 3D), suggesting that the observed phenotype with VU041 is indeed due to Kir channel inhibition (Fig. 3D).

### Knockdown efficiency of irk2 in the fly CNS

**Figure 4:**
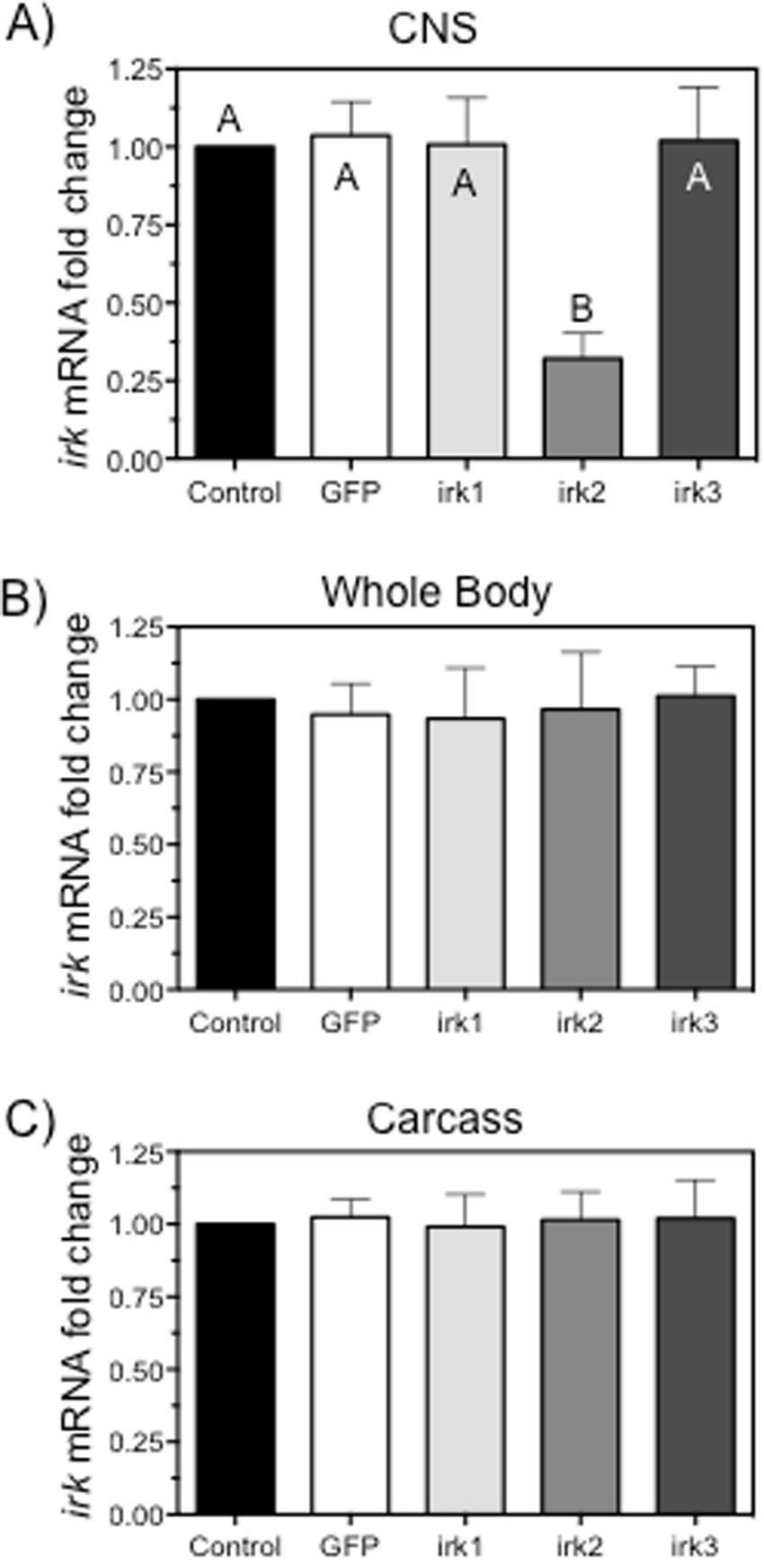
CNS specific RNAi-mediated knockdown of *irk*2. (**A**–**C**) Quantitative RT-PCR analysis of relative mRNA expression levels for *D. melanogaster irk* genes after RNAi-based knockdown in the CNS (**A**), whole body (**B**), and carcass (**C**). Bars represent average (n = 3) fold-difference of *irk* mRNA levels relative to beta-actin control group with error bars representing SEM. Bars not labeled by the same letter represent statistical significance at P < 0.05.

Our data presented in Figs 2 and 3 suggests a critical role of Kir channels in the proper function of the fly nervous system. However, pharmacological probes may modulate physiological pathways outside of the principal target, which raised concerns that a combination of tissues could be responsible for altered neuronal activity after VU041 exposure. To address this concern, we reduced Kir2 mRNA levels specifically in the larval CNS by RNA-interference by using the GAL4-UAS system^28^. Data show the CNS of the F_1_ progeny of *irk*2 knockdown cross expressed 75 ± 11% less *irk*2 mRNA relative to the wildtype (OR) and GFP dsRNA knockdown controls (Fig. 4A). Importantly, relative mRNA levels for *irk*1 and *irk*3 in the CNS were not altered from control flies (Fig. 4A). Furthermore, *irk*2 mRNA levels were not different from the whole body or the carcass of control flies, verifying that the knockdown was CNS specific (Fig. 4B,C).

### Influence of irk2 knockdown to CNS activity and larval movements

**Figure 5:**
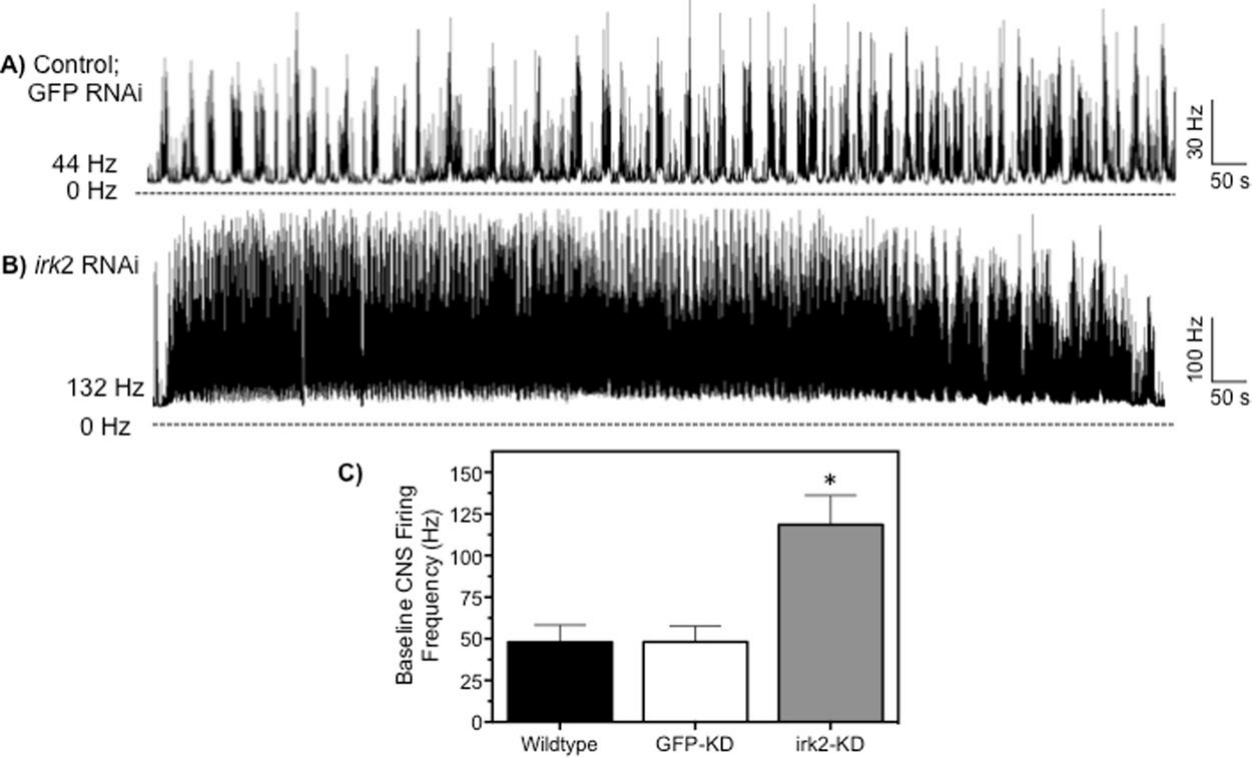
Neurophysiological recordings from the CNS of third instar larvae of *D. melanogaster* after CNS specific knockdown of *irk*2. Representative nerve discharge traces of knockdown control (GFP) flies (**A**) and the *irk*2 knockdown strain (**B**). Initial spike discharge frequencies in spikes/second (Hz) for each experiment are given to the left of each trace. (**C**) Average baseline spike discharge frequency (Hz) of the wildtype (OR) flies, GFP-knockdown (control), and *irk*2 knockdown flies. Bars represent mean (n = 25) spike discharge frequency and error bars represent SEM. *Denotes statistical significance at P < 0.01 as determined by a multiple comparisons test.

Due to the tight regulation of the nervous system, slight modification of ion channel or transporter function is capable of causing significant changes to the function of the nervous system. In line with this notion, the Kir2 knockdown flies were found to have a baseline spike discharge frequency of 118 ± 27 Hz, a 2.6-fold increase when compared to the two control lines. The mean baseline CNS spike discharge frequencies of control and GFP knockdown flies were found to be 48 ± 12 Hz and 49 ± 10 Hz, respectively (Fig. 5C). Importantly, dramatic increase in CNS discharge frequency was also observed in the behavior of the live animal. The maggots with a reduced expression of *irk*2 displayed signs of hyperexcitation reminiscent of maggots that are poisoned with an anticholinergic (e.g. propoxur). Specifically, maggots were observed to move at an increased pace, display uncoordinated head movements, and suffer from consistent twitching of the whole body. The observed phenotype of the live maggots of the knockdown line support the *in vitro* electrophysiological recordings of the CNS shown in Fig. 3. Further, we observed approximately 42% ± 12% reduction in adult emergence from the *irk*2 knockdown flies when compared to control flies, suggesting mortality arose between third-instar and the pupal stage, further supporting the notion that neural Kir channels are critical for survivorship (Figure S1).

### Influence of GPCR- and ATP-gated Kir channel modulators to CNS activity

**Figure 6:**
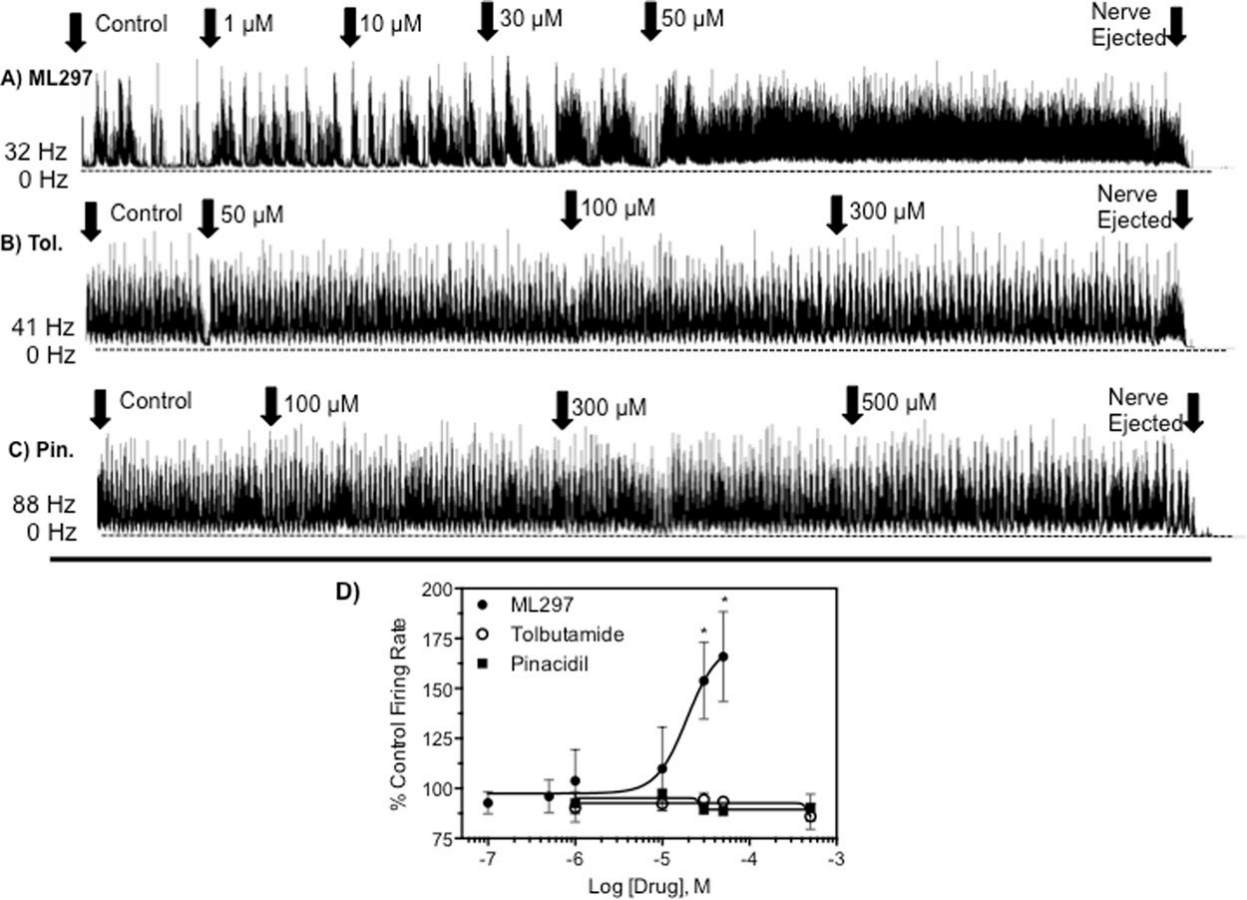
Neurophysiological recordings from the CNS of third instar larvae of *D. melanogaster* after exposure to pharmacological modulators of GIRK and K_ATP_ channels. Representative nerve discharge traces before and after exposure to (**A**) ML297, (**B**) tolbutamide, and (**C**) pinacidil. Initial CNS spike discharge frequencies in spikes/second (Hz) for each experiment are given to the left of each trace. The nerves were ejected from the electrode at the end of the recording to ensure the spike discharge frequency was not noise that had developed throughout the recording procedure. (**D**) Concentration response curve for ML297, tolbutamide, and pinacidil against CNS nerve discharge of *D. melanogaster* larvae from replicated recordings (n = 3–5 concentration per curve, with each concentration replicated at least 5 times), as shown in C. Data points represent mean percentage increase of baseline spike discharge rate, and error bars represent SEM of drug concentrations replicated at least 5 times. When error bars are absent, it is because they are smaller than the size of the symbol. Asterisks represent statistical significance with *representing P < 0.05 as determined by an unpaired t-test to the average baseline spike discharge frequency.

In mammals, there are three functional families of Kir channels that are differentially regulated: (1) ‘classical’ Kir channels that are constitutively active, (2) Kir channels that are regulated by G protein-coupled receptors (GPCRs), which are commonly referred to as G Protein-Coupled inwardly-rectifying potassium channel (GIRK), and (3) ATP-sensitive K^+^ channels (K_ATP_) that are tightly linked to cellular metabolism and are closed in the presence of adenosine triphosphate (ATP). Considering this, we aimed to determine the family or families of Kir channels that is responsible for maintaining proper function of the *Drosophila* CNS. Unfortunately, the pharmacology of GIRK and K_ATP_ channels is nonexistent for insects and is highly underdeveloped for mammals, which limits the scope of the interrogation that can be performed. ML297, a selective activator of mammalian GIRK channels^31,32^, was found to have an excitatory effect to the CNS at mid micromolar concentrations (Fig. 6A). Exposure of the CNS to 30 μM and 50 μM ML297 increased the spike discharge frequency by 53 ± 23% and 63 ± 27%, respectively, when compared to baseline spike discharge frequency, a statistically significant increase (P < 0.05) that was near maximal activation (Fig. 6A,D). Unfortunately, solubility limitations prevented the analysis of higher concentrations and the construction of a full CRC. Lastly, we employed pinacidil and diazoxide (activators) and tolbutamide and glybenclamide (inhibitors) as pharmacological probes to determine the potential for the CNS to be regulated by a K_ATP_ channel. None of the studied K_ATP_ modulators had any influence to the CNS spike discharge frequency at concentrations ranging up to 300–500 μM (Fig. 6B,C, Figure S2).

### Influence of VU041 to resting muscle membrane potential

**Figure 7:**
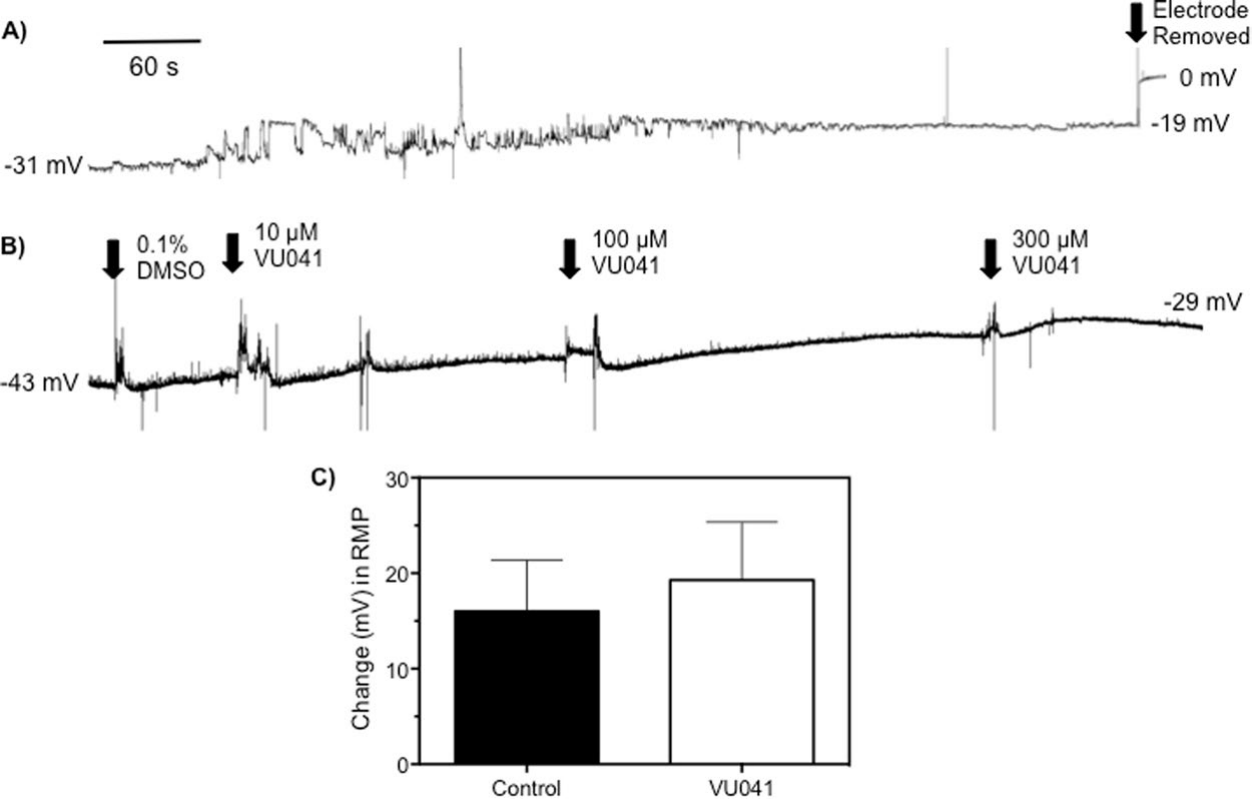
Effects of VU041 on the membrane potential of *D. melanogaster* larval muscle. (**A**) Representative time course trace of membrane potential of *D. melanogaster* muscle bundles before and after exposure to solvent control (DMSO). (**B**) Representative time course trace of membrane potential of *D. melanogaster* muscle bundles before and after exposure to increasing concentrations of VU041. (**C**) Total mV change in resting membrane potential over the 10-minute recording period in control (solvent only) treatments and VU041 treated flies. Bars represent average (n = 10) mV change while error bars represent SEM.

Due to the unique ‘flightless’ phenotype that has been observed in mosquitoes^1,3^ and in *Drosophila* (Fig. 2A), we hypothesized that VU041 is inhibiting Kir channels in the muscle membranes that would lead to inactivation of the muscle. To test this hypothesis, electrophysiological experiments were conducted on insect muscular and neuromuscular (section 3.8) systems using VU041 to investigate the mode of action and putative role of Kir channels in these systems. A representative recording trace is shown in Fig. 7A. Exposure of the body wall muscle sheets to 300 μM VU041 resulted in an average 1.3-fold increase in the resting membrane potential of *D. melanogaster* larval muscle over the course of a 10-minute recording, a non-significant (P = 0.15) increase when compared to control recordings (Fig. 7B).

### Influence of VU041 to neuromuscular junction activity

**Figure 8:**
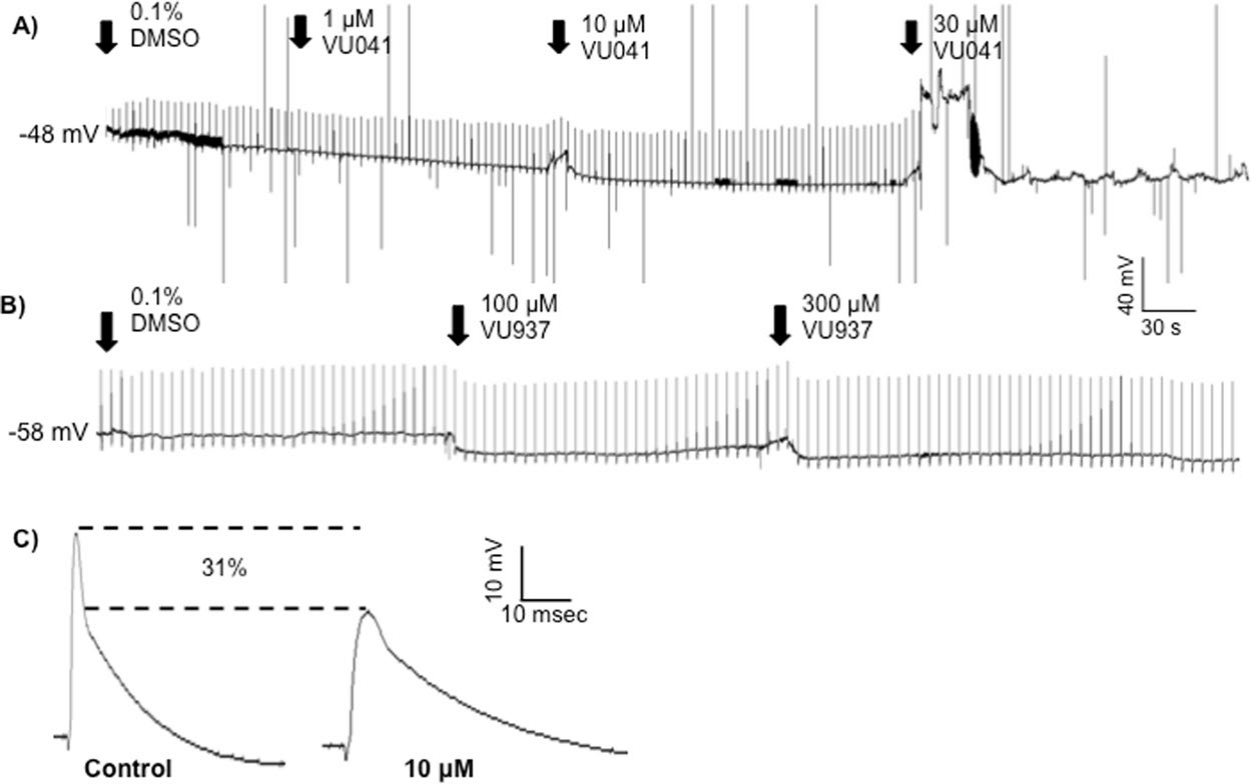
Recordings of the electrically-evoked EPSPs at the neuromuscular junction in *D. melanogaster* larvae after exposure to VU041. (**A**) Representative time course of increasing VU041 concentrations applied to the body wall musculature while recording evoked EPSPs. The remaining transients after block of the EPSP at 30 μM are stimulus artifacts, which are also reflected by any negative excursions from baseline in all traces (artifact amplitudes were truncated from the recordings for clarity of display). The increase in membrane potential after the application of 30 μM is an artifact from the application of the drug and is not a direct response to VU041 since it was not observed in any other recording. (**B**) Representative time course of increasing VU937 concentrations applied to the body wall musculature while recording evoked EPSPs. (**C**) Representative evoked EPSP waveforms after exposure to 10 μM VU041 when compared to control.

Flies that became ‘flightless’ after exposure to VU041 is highly suggestive that Kir channels are responsible for maintaining the physiological makeup of the neuromuscular system, despite the fact that VU041 had no influence to the maintenance of the resting muscle membrane potential. To study the influence of VU041 to the neuromuscular junction (NMJ) we used the dissection preparation described in Swale, *et al*.^24^. At a concentration of 30 μM, VU041 showed a complete and immediate block of the evoked EPSP in the body wall musculature of 3^rd^-instar *D. melanogaster* (Fig. 8A). The complete block was observed at this concentration in all (n = 8) preparations studied. Importantly, no block of the evoked EPSP was observed after exposure to VU937 at concentrations up to 300 μM (Fig. 8B). Although no block of the evoked EPSP was observed at 10 μM VU041, a significant alteration of the evoked EPSP waveform was observed (Fig. 8C). A permanent reduction of the evoked EPSP waveform amplitude was observed with an average (n = 8) reduction of 31 ± 7% when compared to baseline EPSP amplitude, which was a statistically significant (P < 0.05) reduction. Further, the evoked EPSP waveform was broadened by 2.2-fold after exposure to VU041 (10 μM) when compared to baseline spikes with control waveform time course being 61 ± 12 ms and VU041 treated waveforms being 134 ± 16 ms (Fig. 8C), which is a statistically significant increase (P < 0.01). No waveform changes were observed with VU937 when compared to control waveforms.

## Discussion

Currently, there have been no efforts to characterize the physiological role or toxicological potential of insect neural Kir channels. However, our findings demonstrate that the recently discovered Kir-directed insecticide, VU041^3^, is capable of dramatically altering the neural activity of flies and, in a more general sense, that Kir channels constitute a critical K^+^ ion conductance pathway in the insect nervous system. Despite the nervous system being the target tissue of the extreme majority of deployed insecticides^33^, a complete understanding of the physiological pathways critical for proper function of the insect nervous system is still lacking. This represents a critical gap in our knowledge of the complex relationship between the dozens of functionally coupled ion channels, transporters, and enzyme systems that require tight regulation for proper neuronal function. This fundamental gap pertaining to the foundational neural physiology must be filled to develop a holistic understanding of insect nervous system function that will lead to the development of new insecticides.

Knowledge of the physiological role and toxicological potential of insect Kir channels is growing rapidly with studies suggesting these channels serve a critical role in Malpighian tubule function of mosquitoes^17,34–36^ and *Drosophila*^16^, insect salivary gland function^37^, honey bee dorsal vessel function^38^, and insect antiviral immune pathways^39,40^. Furthermore, these channels represent a critical K^+^ conductance pathway in the mammalian nervous system as Kir knockouts in glial cells leads to membrane depolarization, enhanced synaptic potentiation, and reduced spontaneous neural activity^41^. Considering the importance of Kir channels in the function of various insect tissues and the established role of Kir channels in mammalian neuronal tissue, we hypothesized that Kir channels also serve a critical role in insect neural tissue and aimed to highlight the general influence of Kir channel modulation to the insect nervous system through pharmacological and genetic manipulations of the Kir channel.

To begin testing the physiological role of insect neural Kir channels, we performed neurophysiological recordings of the *Drosophila* CNS using the voltage dependent Kir blocker, BaCl_2_. BaCl_2_ is useful pharmacological tool to test the physiological role of Kir channels since, at physiological membrane potentials, Kir channels are up to 1000-fold more sensitive to BaCl_2_ than other K^+^ ion channels^42,43^. This enhanced potency to Kir channels when compared to other K^+^ ion channels enables selective inhibition of Kir channels at low- to mid-micromolar concentrations of BaCl_2_. We observed an increase in the spike discharge frequency followed by cessation of firing after exposure of the CNS to mid-micromolar concentrations of BaCl_2_, providing the first insight that Kir channels constitute a critical conductance pathway in insect CNS. However, the potential for BaCl_2_ to precipitate out of some saline solutions and the potential of BaCl_2_ to modulate non-target proteins limits the conclusions that can be drawn from these data. Fortunately, the recent identification of selective and potent small molecules designed to target insect Kir channels^1–3,18,44^ has facilitated the characterization of the physiological role of these channels in various insect tissue systems with more certainty than BaCl_2_ and other divalent cations. In this study, we used the recently discovered insect Kir channel modulator (VU041) and its inactive analog (VU937)^3^ to characterize the influence these channels have in insect nervous system function. We found that exposure of VU041 to *Drosophila* CNS dramatically altered the spike discharge frequency in a biphasic manner with low concentrations yielding neuroexcitation and higher concentrations having a depressant effect on CNS activity. A biphasic response is oftentimes observed when multiple pathways are inhibited and it is plausible that VU041 is directly or indirectly altering the functional capacity of other ion channels or transporters, such as delayed rectifier K^+^ channels or calcium-activated K^+^ channels. Although off-target effects are possible, they are unlikely since VU937 had no influence to CNS activity, suggesting the observed phenotype is through Kir inhibition. To ensure the observed effect to CNS activity was directly due to Kir2 channel modulation, we performed CNS specific RNAi-mediated knockdown of the Kir2 encoding gene, *irk*2. Results from this genetic depletion of *irk*2 show a dramatic increase in CNS spike discharge frequency that was also substantiated through hyperactive larval behavior. These observed responses to VU041 and *irk*2 genetic depletion is likely due to the physiological role of only Kir2 since no mRNA reduction was observed in other Kir-encoding genes that are expressed in the CNS or any *irk* gene within the whole body or carcass (Fig. 4). Previous reports have documented compensatory functions of Kir channels that arise after genetic depletion of one Kir channel, which prevents the manifestation of an observable change in phenotype^16^. Yet, it does not appear that a compensatory mechanism arose to account for the genetic depletion of *irk*2 since a direct physiological response was observed and *irk*1 and *irk*3 mRNA levels remained unchanged. The influence to expression of other K^+^ ion transport pathways, such as Na^+^-K^+^−2Cl^-^ cotransporter and Na^+^-K^+^-ATPase pumps, remains unknown and should be studied prior to drawing absolute conclusions regarding the physiological basis for neural Kir channels. Furthermore, exposure of the neuromuscular junction to VU041 altered the evoked EPSP waveform and muscle excitability. These data indicate that *Drosophila*, and likely mosquito, central and muscular nervous systems rely on the inward conductance of K^+^ ions through Kir channels for proper function.

The *Drosophila* genome encodes three Kir channel proteins, termed *ir*, *irk*2 and *irk*3^45^, and all three contain the structural features and biophysical properties that are found in mammalian Kir channel subunits. Although *ir* and *irk*3 mRNA has been found to be expressed at low levels in the fly head, the *irk*2 gene is highly expressed in the adult fly head where it is concentrated in the brain and eye^22^, suggesting that, of the Kir channels, *irk*2 is the principal inward conductance pathway for K^+^ ions. The sequence of *irk*2 is similar to that of *ir*, and both are highly related to human Kir 2, 3, and 6 proteins^22,45^, which are constitutively active, GIRK, and ATP-gated Kir channels, respectively. Interestingly, *irk*2 channels have been shown to be constitutively active in S2 cells^45^, associate with sulphonylurea receptors (SUR) as is seen with K_ATP_ channels, and the presence of an Asn223 residue suggests similarity to the GPCR-gated Kirs (Kir3.x; mammalian nomenclature)^22^. The variable functional associations have led to the speculation that *irk*2 may have different mechanisms of gating and regulation based on the cell type the gene is expressed in. Due to this, we employed pharmacological modulators of mammalian GIRK and K_ATP_ channels to determine the mechanisms of *irk*2 gating in the *Drosophila* CNS. The GIRK activator, ML297, is highly selective for mammalian GIRK1/2 subunit combination over other Kir channels^32^ and was found to induce neuroexcitation to the *Drosophila* CNS (Fig. 6A). The sustained increase in *Drosophila* CNS activity after ML297 exposure was unexpected since GIRK2 knockouts in mice revealed an epileptic phenotype, suggesting GIRK is responsible for depressing neuronal excitability and thus, an activator of GIRK should reduce CNS spike discharge frequency^46^. It is important to note that ML297 was shown to have moderate activity on the mammalian serotonin (5-Ht_2b_) receptor^32^, which is expressed in the *Drosophila* CNS^47^ and may be the cause for observed neuroexcitation to the *Drosophila* CNS. Unfortunately, the severely underdeveloped pharmacological library of GIRK inhibitors prevents further interrogation at this time. To determine if *irk*2 is gated by ATP, we employed four structurally distinct activators and inhibitors of mammalian K_ATP_ channels. No change in CNS spike discharge frequency was observed after exposure to these molecules at concentrations ranging into the upper micromolar range. Since other studies have shown clear effects to various insect systems with mammalian K_ATP_ modulators^38–40^, we are confident that the lack of response to the *Drosophila* CNS is due to the absence of ATP-gated Kir channels and not due to incompatibility of the structural scaffolds with the *Drosophila* K_ATP_ channel. These findings have led us to speculate that (1) *irk*2 is not likely to be expressed as a K_ATP_ channel in the CNS, (2) constitutively active Kir channels are present in the *Drosophila* CNS, and (3) GIRK-like channels may be present in the CNS yet further studies are required to interrogate this claim.

The data presented in this study raise the question as to what the physiological role Kir channels have in nervous system function of insects at the cellular level. In mammals, astrocyte function has received significant interest for their roles in the regulation of synaptic levels of neurotransmitters, in particular glutamate, buffering of extracellular K^+^, and release of neurotransmitters, all of which have been shown to directly modulate neuronal excitability and transmission^48,49^. In particular, Kir4.1 channels expressed in astrocytes have been directly linked to K^+^ influx across neural membranes where cells take up excess extracellular potassium ions, distribute them via gap junctions, and extrude the ions at sites in which extracellular K^+^ concentrations ([K^+^]_out_) is low, which is termed K^+^ spatial buffering^41,50–53^. It is reasonable to predict that the insect nervous system employs this method of K^+^ transport during neuronal activity since [K^+^]_out_ is dramatically increased and must be rapidly reversed to prevent membrane depolarization of neurons. Therefore, inhibition of this process through pharmacological blockage of neural Kir channels will lead to depolarization of the nervous system and induce CNS excitation, which was observed in our study at low concentrations of VU041 (Fig. 3) and after genetic knockdown of Kirs (Fig. 5). In mammals, a complete knockout of Kir4.1 yielded a reduction of spontaneous EPSC in pyramidal neurons^41^, similar to what was observed after CNS exposure to concentrations of VU041 greater than 10 μM.

We hypothesize that Kir channels provide a pathway for K^+^ spatial buffering during neuronal activity of *Drosophila* and this pathway is critical for proper CNS activity. Excitability and synaptic transmission of insect and mammalian nervous systems are dependent upon [K^+^]_out_ and alteration of the K^+^ ion gradient directly affects excitatory neurotransmission^54,55^. In accordance to this, we observed changes in the CNS spike frequency and complete cessation of evoked EPSP’s at the NMJ (Figs 3, 5 and 8), which is classically attributed to changes in presynaptic function that may be resultant of altered neurotransmitter release. Similarly, we observed reduced amplitude and broadening of the evoked EPSP waveform at the neuromuscular junction after pharmacological inhibition of Kirs, which may be a result of modification of postsynaptic terminal responsiveness to neurotransmitters^56,57^. The influence of Kir channel inhibition to pre- and post-synaptic function can be due to changes in either extracellular ion or transmitter levels. This is evidenced by the response of the *Drosophila* CNS after exposure to BaCl_2_ and 25 μM VU041. Exposure to these pharmacological agents yielded near maximal spike discharge frequency that culminated in a relatively abrupt termination of this activity. This reduction of CNS spike frequency may be due to depolarization-induced inactivation of Na^+^ channels due to prolonged exposure to elevated [K^+^]_out_, thereby lowering the probability of transmitter release that will reduce neuronal firing^41,58^. Therefore, it appears as though Kir channels are responsible for regulating the K^+^ ion gradient that ultimately controls synaptic activity and neurotransmitter release, which is essential for proper neural signaling and activity.

## Conclusion

Kir channels represent a critical K^+^ ion conductance pathway within the *Drosophila*, and likely mosquito, central and neuromuscular nervous systems. Considering this, it is reasonable to suggest that the recently identified Kir-directed mosquitocide, VU041, is capable of inducing toxicity through neurological poisoning in addition to inducing Malpighian tubule failure that leads to toxicity by an inability to perform osmoregulatory actions^3^. These data provides a proof-of-concept that novel chemical scaffolds targeting neural Kir channels in insects represent a novel mechanism of action with insecticide resistance mitigating potential. Based on the data collected in this study, we hypothesize that the function of Kir channels in the insect nervous system is responsible for reducing [K+]_out_ during neuronal activity by the process known as K+ spatial buffering, similar to that described in mammals^41,59,60^. It is important to note that this hypothesis cannot be fully validated until whole-cell electrophysiological recordings are performed to determine the role of Kirs in (1) glutamate and K^+^ uptake during neural activity, (2) maintenance of neural membrane properties (e.g. V_m_, R_m_, etc), and 3) synaptic transmission and plasticity.

## Electronic supplementary material

Supplemental Information
