# Supplementary material for: Inwardly Rectifying Potassium (Kir) Channels Represent a Critical Ion Conductance Pathway in the Nervous Systems of Insects
Source: Sci Rep. 2018 Jan 25;8:1617. doi: 10.1038/s41598-018-20005-z (PMC5785497; doi:10.1038/s41598-018-20005-z)
Supplement: Supplementary file 1 — Supplemental Information [file 41598_2018_20005_MOESM1_ESM.pdf]

Supplemental Information

Inwardly Rectifying Potassium (Kir) Channels Represent a Critical Ion Conductance Pathway in the Nervous Systems of Insects

Authors: Rui Chen[1], Daniel R. Swale[1*]

Author Affiliations:

[1] Louisiana State University AgCenter, Department of Entomology, Baton Rouge, LA 70803

Supplemental Methods

Adult emergence

Fifteen adult female and 5 adult male *Drosophila* flies were released into a *Drosophila* vial containing standard medium and were maintained at 25°C, 12 hour-12 hour photoperiod and 55% relative humidity. Flies were removed from the vial 72 hours after being released, which was ample time to ensure oviposition occurred. Adult emergence was determined by counting the number of adult flies that emerged daily for 28 days after the removal of the parental flies. After the daily count, flies were removed to prevent recounting of flies. Total emergence was performed on five replicates for the control (OR), GFP knockdown (knockdown control), and *irk*2 knockdown flies. Percent emergence was determined through comparison of GFP and irk2 knockdown emergence to emergence of OR fly strain, which was standardized to unity. Data were plotted using GraphPad Prism (La Jolla, CA) software. Statistical analysis was performed using an unpaired *t*-test of the RNAi strains compared to OR.

Neurophysiological recordings of Drosophila *CNS*

The methods for determining the influence of diazoxide and glybenclamide were the same for the other electrophysiological recordings of the CNS, which is described in section 2.4.

Supplemental Figures

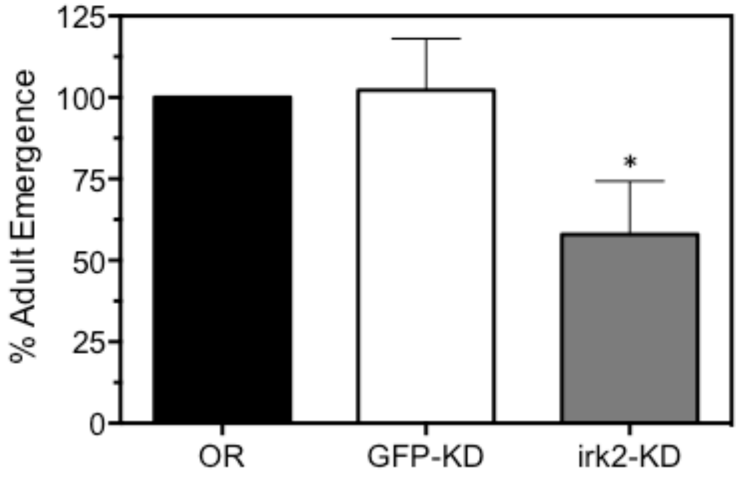

Figure S1. **Percent adult emergence of *irk*2 RNAi-knockdown flies.** Bars represent average (n=5) percent emergence of control (GFP) and *irk*2 RNAi-mediated knockdown when compared to OR flies. Error bars represent SEM. Asterisks represent statistical significance at P<0.05.

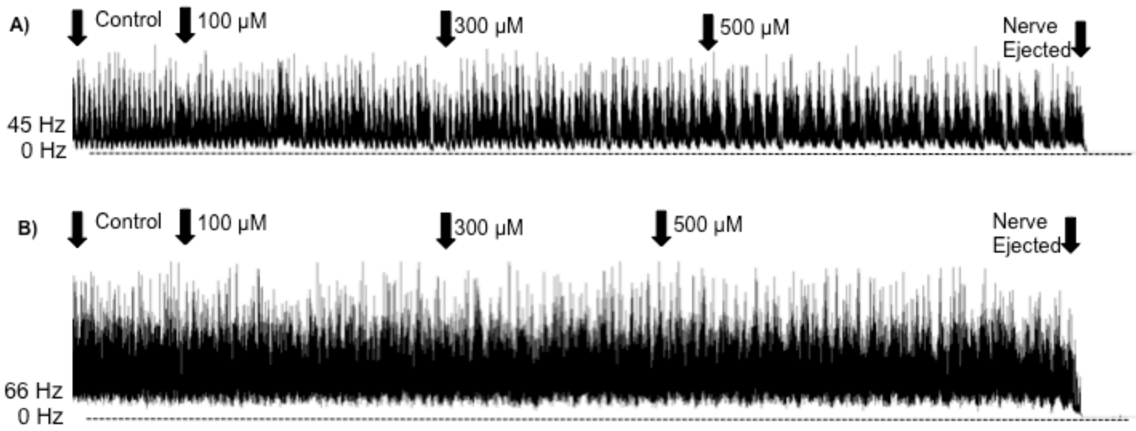

Figure S2. **Neurophysiological recordings from the CNS of third instar larvae of *D. melanogasgter* after exposure to K_{ATP} modulators.** Representative nerve discharge traces of the K_{ATP} opener diazoxide (A) and the K_{ATP} inhibitor glibenclamide (B). Initial firing frequencies in spikes/second (Hz) for each experiment are given to the left of each trace.
